# Effects of Stevia on Inflammatory Markers, Renal and Hematological Parameters in Patients With Stage I-III Chronic Kidney Disease: A Randomized, Placebo-Controlled Clinical Trial in Bangladesh

**DOI:** 10.7759/cureus.88152

**Published:** 2025-07-17

**Authors:** Farhana Rizwan, Saquiba Yesmine, Harun Ur Rashid, Forhad Monjur, Mamunur Rahman, Tapan Kumar Chatterjee

**Affiliations:** 1 Department of Pharmacy, East West University, Dhaka, BGD; 2 Department of Pharmaceutical Technology, Jadavpur University, Kolkata, IND; 3 Department of Pharmacy, Jahangirnagar University, Dhaka, BGD; 4 Department of Nephrology, Kidney Foundation Hospital and Research Institute, Dhaka, BGD; 5 Department of Hematology and Clinical Pathology, Kidney Foundation Hospital and Research Institute, Dhaka, BGD; 6 Department of Pharmaceutical Science and Technology, JIS University, Kolkata, IND

**Keywords:** angiotensin-ii receptor blocker (arb), ca2+ channel blocker (ccb), chronic kidney disease (ckd), diabetes mellitus, erythrocyte sedimentation rate (esr), high-sensitive c-reactive protein (hscrp), hypertension, renoprotective effects, stevia

## Abstract

Background

Global public health policy is concerned about the increasing prevalence of chronic kidney disease (CKD) and its associated comorbidities. Stevioside has been confirmed by food regulatory and safety bodies to be safe and effective for treating diabetes and hypertension. Stevia has also demonstrated a nephroprotective effect in experimental animals. This clinical trial aimed to investigate the kidney-protective effects of stevioside in CKD Stage I-III patients, and to explore its impact on inflammatory markers, kidney function, and hematological parameters as a potential new treatment option.

Methods

The trial was a prospective, single-blind, placebo-controlled study with 93 participants (83 CKD patients at Stage I-III and 10 healthy controls). The 83 CKD patients were randomly assigned to stevia (250 mg twice daily) or placebo (in addition to standard care), and 10 healthy individuals served as a control group. The study was conducted at the Kidney Foundation Hospital and Research Institute, Dhaka, Bangladesh, from December 2016 to December 2018. Patients were scheduled for follow-up visits every three months for a total of nine months. Data were collected using a systematic, validated, and structured questionnaire.

Results

At baseline, 44.2% of the CKD patients were in Stage III; by the second follow-up, this proportion had dropped to 38.2%. Stevia treatment significantly reduced systolic blood pressure (p < 0.043), diastolic blood pressure (p < 0.001), microalbuminuria (p < 0.003), postprandial blood sugar (p < 0.001), erythrocyte sedimentation rate (p < 0.023), and high-sensitivity C-reactive protein (p < 0.007) levels at the second follow-up. During the washout period (with no stevia), most of these improved values trended back toward baseline in the stevia group, indicating a loss of the treatment effect upon withdrawal.

Conclusion

This nine-month clinical investigation found that oral stevia can positively impact biochemical indicators in CKD patients, potentially mitigating the progression of the disease. Therefore, stevia’s benefits might offer new interventions to alleviate cardiovascular and metabolic risks in early-stage CKD patients.

## Introduction

The global burden of chronic kidney disease (CKD) is immense. The prevalence of CKD is predicted to increase to 35% in older people over 60 in the USA [1], 12.9% in Japan [2], and 13.0% in China [3]. Several studies have reported an increasing prevalence of CKD among Bangladeshi adults, and the overall prevalence was 17.3% [4,5]. The Third National Health and Nutrition Examination Survey (NHANES III) identified low socioeconomic status, male sex, adult age, diabetes, high blood pressure, and environmental factors as significant predictors of CKD, especially in developing nations of South Asia [6-8].

Stevioside is a glycoside isolated from the plant *Stevia rebaudiana* Bertoni and has been widely used as a sweetening agent in Japan for more than 20 years [9]. Steviol and its derivatives - isosteviol, dihydroisosteviol, and 16-oxime isosteviol - are metabolic products of intestinal microflora, which the stomach can readily absorb, allowing them to enter the bloodstream and travel to the kidneys [10,11]. Stevioside has been reported to have anti-hyperglycemic, anti-hypertensive, anti-inflammatory, anti-tumor, anti-diarrheal, and immune-regulating properties [10]. Stevioside's potential mechanisms of action against CKD are currently being studied. 

The inflammatory process plays an integral part in the development of atherosclerosis. On the other hand, CKD shares similar pathophysiological pathways with atherosclerosis. Published reports have shown that oxidative stress and inflammatory indicators contribute to the onset of diabetes mellitus and hypertension, both of which are possible risk factors for CKD [12]. Significantly higher levels of inflammatory markers among CKD patients have strengthened the correlation with changes in estimated glomerular filtration rate (eGFR), as documented by several studies [13]. However, the link between inflammatory indicators and the likelihood of developing CKD in humans remains unclear [14]. There is evidence suggesting that some undetermined inflammatory processes contribute to end-stage renal disease (ESRD) [15]. Since inflammation plays a major role in CKD progression, and stevioside is anti-inflammatory, investigating its effects in CKD patients might provide new avenues for disease management.

As such, the present interventional, randomized controlled trial investigated the effects of stevia on inflammatory markers, as well as renal and hematological parameters, in CKD patients at Stage I-III. To our knowledge, this is the first long-term clinical trial conducted on CKD patients. A preliminary report of the baseline and first follow-up results of this trial has been published previously [16]. In contrast, the current manuscript presents the full nine-month follow-up data - including the second follow-up (at six months) and the washout period - along with an analysis of inflammatory markers, thereby providing novel insights beyond the initial findings. The primary objective of this trial was to evaluate whether stevia (stevioside) supplementation offers renoprotective effects in early-stage CKD (Stage I-III) by assessing its impact on inflammatory markers, kidney function, and hematological parameters compared to placebo.

## Materials and methods

Study design

This study was a prospective, interventional, single-blind, placebo-controlled, washout clinical trial conducted at the Kidney Foundation Hospital and Research Institute, Dhaka, Bangladesh, from December 2016 to December 2018. A total of 93 participants were included. Among them, 83 adult CKD patients (Stage I-III) comprised the treatment cohort, and 10 healthy individuals served as a control group. The 83 CKD patients were randomly assigned in parallel to one of two groups: Stevia (STV, n = 43) or Placebo (PLC, n = 40). Eighty-three CKD patients were randomly assigned (1:1) to the STV or PLC group using a computer-generated sequence. We did not employ block randomization or stratify by any baseline characteristics; instead, an independent statistician generated a fully random allocation sequence.

This was a single-blind trial, meaning that the participants were blinded to whether they were receiving stevia or placebo, while the investigators and care providers were aware of the assignment. Laboratory personnel conducting the assays were not explicitly informed of group allocation. In addition to their assigned intervention, all CKD patients continued their standard care, including antihypertensive (e.g., angiotensin receptor blockers (ARBs) or angiotensin-converting enzyme (ACE) inhibitors) and antidiabetic medications, as appropriate. Participants in the STV group received capsules containing 250 mg of stevioside (stevia) twice daily, while those in the PLC group received matching placebo capsules twice daily.

The intervention period lasted six months. After six months of supplementation, there was a planned washout period of three months (months 7-9), during which no stevia was given, to evaluate the persistence of any effects after cessation of the treatment. The three-month duration was deliberately chosen on the basis of a number of clinical and methodological considerations. It is known that measurable changes in renal function, blood pressure, and inflammatory markers over time in patients with early-stage CKD are gradual and subtle. Thus, the overall study duration for each participant was nine months from baseline.

The study protocol was approved by the Human Research Ethics Committee of the Kidney Foundation Hospital and Research Institute (Ethical Approval Registration No: KFHRI/ECC-001/2016). The trial was registered on January 1, 2016, in the institutional clinical trials registry of the Kidney Foundation Hospital and Research Institute, Bangladesh (accessible at the hospital’s website). The study methodology adhered to the ethical standards of the 1989 amendments to the Declaration of Helsinki. Institutional Review Board (IRB) oversight was in place to ensure compliance with ethical guidelines (CPMP/ICH/135/95). All participants provided written informed consent before enrollment. This trial was designed as a superiority trial to determine if stevia provides greater renoprotective effects than placebo.

Study participants

Adults aged 31 to 70 with Stage I to III CKD, both male and female, were included in the study. Participants who had a myocardial infarction, undergone coronary artery bypass grafting, experienced a cerebrovascular accident, undergone coronary angioplasty, had a transient ischemic attack, had any history of heart failure prior to enrollment, or had morbid obesity or chronic sepsis were excluded [16]. Initially, 105 participants were enrolled in the study, of whom 93 successfully completed the entire study period and requirements. The case and control groups were matched by age (details are given in the previously published part of this study) [16]. Participants in this study were randomly assigned to either the STV group or the PLC group through a computer-generated auto-sequence. Participant allocation was sealed using a paper envelope. The randomization order was assigned by an expert statistician who was not involved in the study, in order to avoid bias [16].

Study procedures

Baseline data were collected at enrollment (month 0) for all participants via a structured questionnaire and clinical examination (see Appendix). Follow-up assessments for CKD patients in the STV and PLC groups were conducted at three months (first follow-up) and six months (second follow-up) during the intervention, and again at nine months (end of washout). Healthy controls (CON) underwent baseline and periodic assessments at similar time points for comparison, although they received no intervention [16].

At each visit, clinical measurements (e.g., blood pressure, body weight, and BMI) and biochemical parameters were evaluated. Fasting and postprandial blood sugar (PBS) levels were measured, along with kidney function tests (serum creatinine, blood urea, eGFR calculation) and urinary markers (microalbumin, urinary total protein, albumin-to-creatinine ratio, or ACR). Inflammatory markers, specifically high-sensitivity C-reactive protein (hsCRP) and erythrocyte sedimentation rate (ESR), were measured to assess systemic inflammation.

These tests were performed in the hospital’s biochemical and hematological laboratory using standardized methods and kits. All laboratory assays were quality-controlled and, when applicable, blinded to the intervention assignment. Adherence to the stevia or placebo regimen was monitored by capsule counts and patient self-reports at each visit. Participants were also asked to maintain their usual diet and exercise habits during the trial and to report any new symptoms or adverse events.

The study staff closely monitored for any potential side effects of stevia (none were anticipated beyond mild gastrointestinal upset, and none were reported). Compliance was high; all study participants adhered to the prescribed treatment schedule for the entire nine-month period, and compliance was comparable across the STV, PLC, and CON groups.

Sample size calculation

The study sample size was calculated by the following formula: $n = \frac{2[(a + b)^2 \sigma^2]}{(\mu_1 - \mu_2)^2}$, where n is the sample size in each of the groups, μ_1_ and μ_2_ are the population means in treatment groups 1 and 2, respectively, σ² is the population variance (standard deviation), a is the conventional multiplier for alpha (α = 0.05), and b is the conventional multiplier for power (0.80). The study power was considered to be 80% (0.80) [16,17].

Data management and statistical analysis

Statistical analysis was performed using IBM SPSS Statistics for Windows, Version 23 (released 2015; IBM Corp., Armonk, NY, USA). In the descriptive analysis, continuous variables were expressed as mean ± standard deviation (SD), and categorical variables were expressed as percentages (%). The data were analyzed using one-way analysis of variance (ANOVA), followed by Bonferroni’s test (for multiple comparisons), regression analysis, and an independent-samples t-test. Variables with p-values less than 0.05 were considered statistically significant. Baseline differences among the three groups (STV, PLC, and CON) were analyzed using one-way ANOVA with Bonferroni post hoc tests, since there were more than two groups. In contrast, outcome differences between the two patient groups (STV vs. PLC) at each follow-up point were assessed using independent-samples t-tests. A linear regression analysis was conducted with baseline eGFR as the dependent variable and various urinary markers (ACR, protein-to-creatinine ratio (PCR), and microalbumin levels across the treatment phases) as independent variables.

## Results

**Figure 1:**
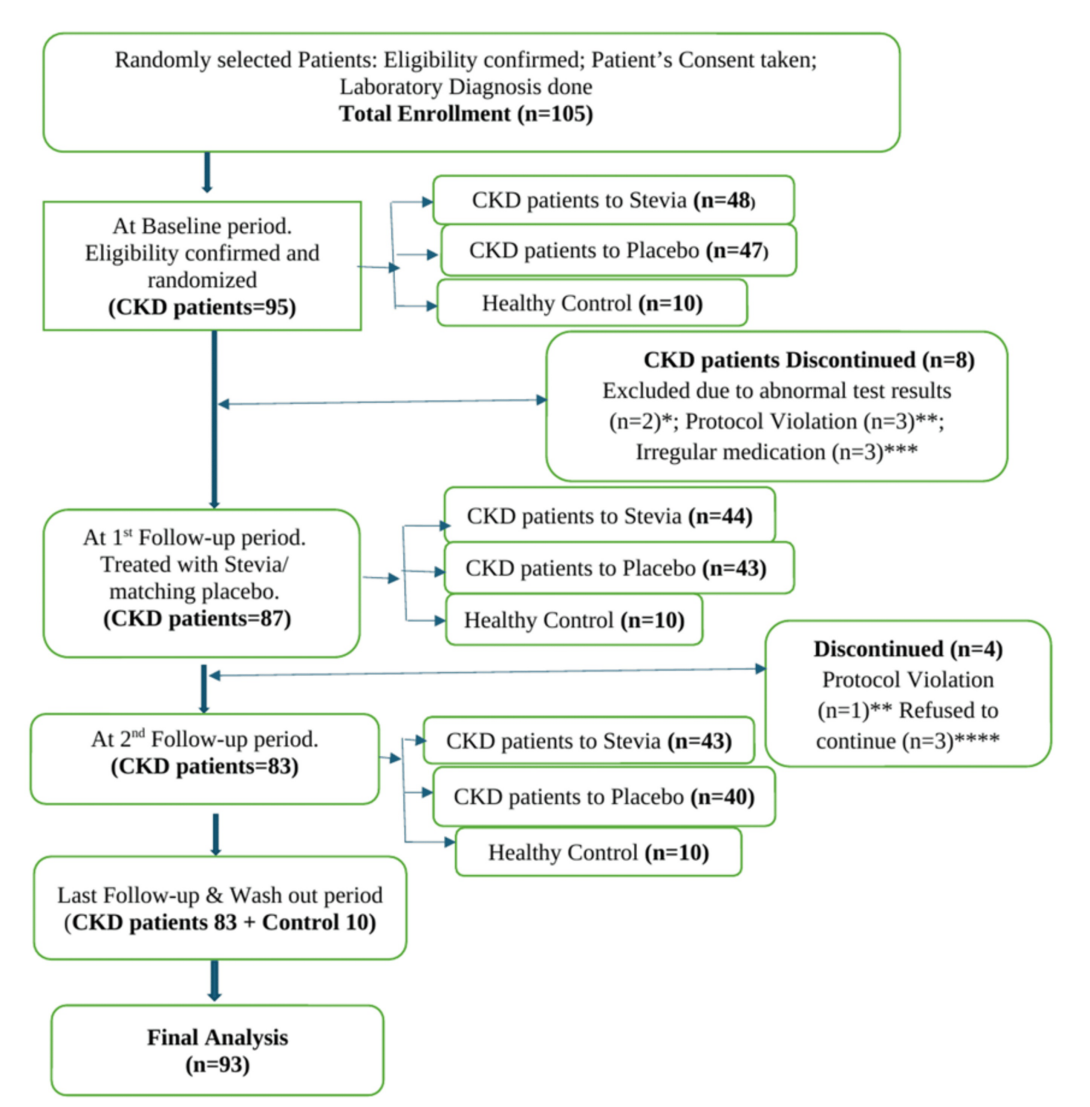
Patient disposition Here, the first follow-up represents the assessment after three months from the baseline period, the second follow-up signifies the evaluation after three months from the first follow-up, and the washout period indicates the assessment after three months from the second follow-up. *Patients were excluded at the baseline period because laboratory results did not meet the inclusion criteria; **Patients were discontinued because of protocol violations at baseline and the first follow-up period, as identified by the principal investigator; ***Patients were discontinued due to irregular medication intake during the baseline period; ****Patients refused to continue the trial at the first follow-up period due to personal reasons. CKD, Chronic Kidney Disease

In this interventional clinical study, we measured the effect of stevia on the early stages of CKD. We compared changes in biometric parameters, urinary variables, and hematological parameters across different treatment phases - baseline, first follow-up, second follow-up, and washout period - among 93 participants. Of these 93 participants, 83 were CKD patients (Stage I to III), with 43 in the STV group and 40 in the PLC group; 10 healthy participants were assigned to the CON group, and their data were analyzed. Initially, 105 participants fulfilled the inclusion criteria and were enrolled in this trial, as shown in Figure 1. Based on laboratory analysis, 95 CKD patients were randomized, and 10 healthy adults were assigned to the control group. After the baseline phase, eight patients - four from the STV group and four from the PLC group - discontinued for various reasons. A total of 97 participants (87 CKD patients and 10 healthy controls) returned for the first follow-up. However, four patients - one from the STV group and three from the PLC group - discontinued after the first follow-up. A total of 83 CKD patients in Stage I to III continued through the second follow-up and the washout period (Figure 1). The demographic factors and preliminary investigation reports from the baseline and first follow-up phases were previously published in a separate article [16].

Our previous article showed that the mean age of the participants was STV = 55 ± 11.75, PLC = 53.6 ± 11.27, and CON = 47.20 ± 4.87 years, respectively, of whom 53.61% were male; their group-wise distribution was STV = 52.3%, PLC = 55.8%, and CON = 50%, respectively [16]. Married participants comprised 84.5% (STV = 84.1%, PLC = 95.3%, CON = 40%); widowed, 8.2%; and single, 7.2%. We found that 48.46% of the participants were from Dhaka city, and the rest (51.54%) were from outside Dhaka. Cigarette smokers were found to be 47.1% (STV), 44.4% (PLC), and 8.8% (CON). Among the participants, STV = 44.2% and PLC = 55.8% were Stage-III CKD patients; STV = 54.5% and PLC = 36.4% were in Stage II; and STV = 35.3% and PLC = 23.5% were in Stage I [16]. The randomly selected clinically stable CKD patients had either systemic hypertension, diabetes, or both. The group-wise random distribution of CKD patients taking ARBs was STV (61.2%) and PLC (65.1%); calcium channel blockers (CCBs) were STV (40.9%) and PLC (37.2%); and a combination of ARB and CCB was STV (2.3%) and PLC (2.3%). Of the total participants, 25.4% were taking antidiabetic medication; of these, 63.6% were in the STV group, and 67.4% were in the PLC group [16].

Stevia treatment improved eGFR, as demonstrated by the number of Stage III patients, which steadily decreased after the first and second follow-up screenings (baseline = 44.2%, first follow-up = 39.4%, second follow-up = 38.2%). However, the number of Stage III patients increased again to 40.5% during the washout period, when no stevia was given (Table 1). At the end of the six-month treatment period, a slight decrease in BMI was observed up to the second follow-up phase of stevia treatment. However, no significant changes were observed in body weight or BMI after the nine-month study period (Table 2). After three and six months of treatment, a fall in blood pressure readings was seen in both the STV and PLC groups (Table 2). The drop in blood pressure began after seven days and persisted throughout the entire treatment period, according to patients’ self-monitoring records (records not shown here). After stevia administration, the second follow-up revealed a significant reduction in diastolic blood pressure (DBP) (80.46 ± 0.57 mmHg; p < 0.001) and a mean drop in systolic blood pressure (SBP) (118.13 ± 1.16 mmHg), though the latter was statistically non-significant. Fasting blood sugar (FBS) and PBS levels were also reduced in the STV group. PBS dropped significantly (p < 0.001) by six months, whereas changes in the PLC group were smaller. This aligns with stevia’s reported anti-hyperglycemic effect, as shown in Table 2.

**Table 1:** Status of CKD stages among patients receiving treatment at different intervention phases Descriptive analysis was performed to determine the different stages of CKD among the patients. No control group was included in this table. The different stages of CKD are as follows: Stage I = eGFR 90 mL/min/1.73 m² or higher; Stage II = eGFR 60 to 89 mL/min/1.73 m²; and Stage III = eGFR 30 to 59 mL/min/1.73 m², respectively. STV, Stevia Treatment Group; PLC, Placebo Group; BL, Baseline Phase; 1st FF, 1st Follow-Up Phase; 2nd FF, 2nd Follow-Up Phase; WO, Washout Phase; CKD, Chronic Kidney Disease; eGFR, Estimated Glomerular Filtration Rate

Stages of CKD	STV (n = 43)				PLC (n = 40)			
	BL	1st FF	2nd FF	WO	BL	1st FF	2nd FF	WO
Stage I	6 (35.3%)	6 (30%)	7(33.3%)	6 (37.5%)	4 (23.5%)	7 (35%)	7 (33.3%)	3(18.8%)
Stage II	18 (54.5%)	24 (60%)	23 (60.5%)	22 (55.0%)	12 (36.4%)	13 (32.5%)	12 (31.6%)	15 (37.5%)
Stage III	19 (44.2%)	13 (39.4%)	13 (38.2%)	15 (40.5%)	24 (55.8%)	20 (60.6%)	21 (61.8%)	22 (59.5%)

**Table 2:** Comparison of various physiological and biochemical indicators of the participants in different treatment phases The values were presented as mean (±SEM). Data was analyzed by an independent sample t-test. The stevia treatment group is expressed as STV (n = 43); the placebo treatment group is expressed as PLC (n = 40); and the healthy control group is expressed as CON (n = 10). Here, (*p < 0.05) = significant, (**p < 0.01) = highly significant, and is considered as STV vs PLC. Data are presented as mean ± SD. Analysis: One-way ANOVA was used for comparisons across the three groups, with post-hoc Bonferroni correction. Independent t-tests were used for direct comparisons between two groups. Significant p-values are indicated with symbols (p < 0.05, etc.). BL, Baseline Phase; 1st FF, 1st Follow-Up Phase; 2nd FF, 2nd Follow-Up Phase; WO, Washout Phase; SBP, Systolic Blood Pressure; DBP, Diastolic Blood Pressure; FBS, Fasting Blood Sugar; PBS, Postprandial Blood Sugar; STP, Serum Total Protein; UTP, Urinary Total Protein; ACR, Albumin-to-Creatinine Ratio; PCR, Protein-to-Creatinine Ratio; eGFR, Estimated Glomerular Filtration Rate; ESR, Erythrocyte Sedimentation Rate; HsCRP, High-Sensitivity C-Reactive Protein

Physiological & Biochemical Variables		STV				PLC				CON			
		BL	1st FF	2nd FF	WO	BL	1st FF	2nd FF	WO	BL	1st FF	2nd FF	WO
Blood Pressure	SBP (mmHg)	133.72 (±3.3); p = 0.054	125.34 (±2.00); p = 0.595	118.13 (±1.16); p = 0.043	131.74 (±1.52); p = 0.501	134.12 (±2.73); p = 0.076	123 (±2.40); p = 0.327	127.25 (±1.60); p = 0.577	134.17 (±1.90); p = 0.719	111 (±3.48)	111 (±3.14)	111 (±3.14)	111 (±3.14)
	DBP (mmHg)	84.88 (±1.95); p = 0.126	79.30 (±0.84); p = 0.081	80.46 (±0.57); *p = 0.001	87.67 (±1.09); p = 0.397	82.50 (±1.22); p = 0.587	78.37 (±1.66); p = 0.492	86.25 (±1.74); p = 0.174	86.87 (±0.99); p = 0.171	79 (±3.14)	76 (±2.21)	76 (±2.21)	79 (±3.14)
Electrolytes	Na (mEq/L)	138.55 (±0.46); p = 0.113	143.95 (±4.7); p = 0.114	140.02 (±0.33); p = 0.722	139.6 (±0.35); p = 0.942	138.82 (±0.38)	140.12 (±0.40)	140.1 (±0.31)	139.50 (±0.34)	139.9 (±0.37)	140.02 (±0.44)	140 (±0.44)	139.9 (±0.37)
	K (mEq/L)	3.90 (±0.07); p = 0.456	3.98 (±0.07); p = 0.692	3.85 (±0.06); p = 0.475	3.99 (±0.07); p = 0.094	4.17 (±0.07)	4.10 (±0.08)	4.04 (±0.06)	4.25 (±0.06)	4.24 (±0.05)	4.05 (±0.07)	4.05 (±0.07)	4.24 (±0.05)
	Cl (mEq/L)	102.51 (±0.65); p = 0.51	103.53 (±0.50); p = 0.369	104.15 (±0.35); p = 0.903	102.79 (±0.52); p = 0.043	103.30 (±0.51)	105.20 (±0.44)	103.7 (±0.39)	104.22 (±0.41)	103.7 (±0.36)	105.1 (±0.79)	105.1 (±0.79)	103.7 (±0.36)
	TCO_2_ (mEq/L)	27.39 (±0.29); p = 0.486	27.23 (±0.034); p = 0.972	27.37 (±0.30); p = 0.718	28.13 (±0.23); p = 0.101	26.76 (±0.28)	26.75 (±0.38)	26.62 (±0.36)	27.42 (±0.34)	26.1 (±0.10)	26.8 (±0.32)	26.8 (±0.32)	26.1 (±0.10)
Inorganic Phosphate (mg/dL)		1.29 (±0.10); p = 0.054	1.17 (±0.03); p = 0.824	1.24 (±0.03); p = 0.730	1.43 (±0.10); p = 0.135	1.09 (±0.03)	1.12 (±0.03)	1.13 (±0.03)	1.19 (±0.03)	1.14 (±0.04)	1.14 (±0.05)	1.14 (±0.05)	1.14 (±0.04)
Ca (mmol/L)		3.23 (±0.63); **p = 0.009	2.18 (±0.02); p = 0.876	2.18 (±0.01); *p = 0.025	2.53 (±0.19); *p = 0.026	2.23 (±0.02)	2.19 (±0.02)	2.21 (±0.01)	2.32 (±0.02)	2.30 (±0.02)	2.26 (±0.03)	2.26 (±0.03)	2.30 (±0.02)
Blood Sugar Level	FBS (mmol/L)	6.93 (±0.34); p = 0.182	6.70 (±0.29); p = 0.337	5.98 (±0.31); p = 0.069	7.09 (±0.28); p = 0.506	6.32 (±0.22)	6.98 (±0.40)	6.86 (±0.38)	6.53 (±0.22)	5.53 (±0.15)	5.05 (±0.26)	5.05 (±0.26)	5.53 (±0.15)
	PBS (mmol/L)	9.54 (±0.57); p = 0.162	9.24 (±0.49); p = 0.066	7.62 (±0.24); *p = 0.000	9.66 (±0.42); *p = 0.000	9.42 (±0.76)	10.07 (±0.72)	9.7 (±0.60)	10.78 (±0.70)	6.34 (±0.39)	6.08 (±0.31)	6.08 (±0.31)	6.34 (±0.39)
Urinary Variables	Blood Urea (mmol/L)	8.33 (±1.26); *p = 0.004	5.86 (±0.68); p = 0.233	5.30 (±0.16); p = 0.147	6.9 (±0.76); p = 0.150	5.48 (±0.63)	5.2 (±0.22)	4.92 (±0.20)	5.38 (±0.19)	3.2 (±0.29)	3.4 (±0.45)	3.4 (±0.45)	3.2 (±0.29)
	Se. Creatinine (µmol/L)	1030.70 (±3.38); p = 0.268	101.07 (±3.64); p = 0.054	98.49 (±3.5); p = 0.251	101.11 (±4.13); p = 0.478	104.88 (±4.67)	110.76 (±4.87)	108.57 (±4.49)	108.75 (±3.74)	70.9 (±3.49)	71 (±3.2)	71.43 (±3.2)	70.9 (±3.49)
	Se. uric Acid (mg/dL)	362.25 (±20.77); p = 0.194	341.09 (±16.41); p = 0.895	306.32 (±12.81); p = 0.991	355.11 (±13.08); p = 0.559	313.72 (±16.89)	351.25 (±16.60)	381.4 (±14.27)	341.62 (±14.55)	368 (±20.77)	364 (±37.18)	364 (±37.18)	368 (±34.21)
	STP (gm/dL)	71.86 (±1.81); p = 0.058	71.30 (±1.65); p = 0.102	76.18 (±0.77); p = 0.275	74.10 (±1.86); p = 0.019	74.40 (±0.88)	74.22 (±0.77)	74.10 (±1.86)	74.32 (±0.75)	71.6 (±1.81)	71.9 (±1.78)	71.9 (±1.78)	71.6 (±1.15)
	Microalbumin (SPOT) (mg/dL)	129.03 (±40.15); *p = 0.001	172.07 (±46.67); *p = 0.001	121.42 (±35.31); *p = 0.003	151.6 (±42.36); *p = 0.001	195.22 (±41.83)	172.29 (±40.15)	139.62 (±34.19)	195.82 (±39.14)	10 (±1.39)	5.9 (±0.86)	5.9 (±0.86)	4.7 (±1.39)
	UTP (SPOT) (mg/dL)	48.95 (±12.31); p = 0.784	71.03 (±19.27); p = 0.148	72.62 (±16.94); p = 0.005	64.41 (±16.06); p = 0.348	58.97 (±12.37)	56.67 (±12.74)	45.75 (±8.78)	61.88 (±12.22)	8.20 (±1.61)	15.9 (±6.67)	15.9 (±6.67)	8.2 (±1.61)
	ACR (mg/g)	184.37 (±63.50); p = 0.615	184.40 (±52.64); p = 0.348	169.53 (±50.08); p = 0.662	176.31 (±59.90); p = 0.155	235.88 (±57.41)	168.26 (±42.93)	172.29 (±44.72)	316.81 (±67.37)	5.74 (±1.10)	5.98 (±1.23)	5.98 (±1.23)	5.74 (±1.10)
	PCR (mg/g)	65 (±0.14); p = 0.638	10.96 (±9.70); p = 0.050	10.24 (±9.70); p = 0.058	0.651 (±0.13); *p = 0.000	14 (±0.14)	0.51 (±0.10)	0.53 (±0.09)	2.52 (±0.41)	0.11 (±0.02)	0.15 (±0.05)	0.15 (±0.05)	0.11 (±0.02)
	eGFR (mL/min/1.73m^2^)	64.02 (±3.25); p = 0.377	66.27 (±3.34); p = 0.999	68.02 (±3.26); p = 0.553	65.39 (±3.29); p = 0.326	61.24 (±2.77)	61.05 (±3.23)	60.91 (±2.98)	60.94 (±2.61)	104.8 (±4.71)	103.6 (±4.97)	103.6 (±4.97)	104.8 (±4.71)
Hematological Variables	Hb (g/dL)	12.48 (±0.24); p = 0.510	12.54 (±0.23); p = 0.929	13.33 (±0.19); p = 0.165	12.63 (±0.25); p = 0.584	12.55 (±0.24)	12.62 (±0.26)	12.75 (±0.25)	12.57 (±0.25)	13.43 (±0.68)	13.16 (±0.69)	13.16 (±0.69)	13.43 (±0.68)
	RBC (McL)	4.50 (±0.07); p = 0.816	4.63 (±0.09); p = 0.945	4.62 (±0.09); p = 0.626	4.58 (±0.07); p = 0.925	4.63 (±0.08)	4.69 (±0.09)	4.75 (±0.08)	4.65 (±0.08)	4.80 (±0.14)	4.68 (±0.15)	4.68 (±0.15)	4.8 (±0.14)
	WBC (PermicroL)	9.35 (±0.42); p = 0.317	8.89 (±0.35); p = 0.553	8.92 (±0.31); p = 0.771	9.40 (±0.41); p = 0.302	8.82 (±0.42)	8.70 (±0.32)	8.85 (±0.30)	8.81 (±0.30)	7.38 (±0.48)	7.06 (±0.51)	7.06 (±0.51)	7.38 (±0.48)
	Platelet (PermicroL)	311.9 (±11.7); p = 0.576	302.74 (±12.76); p = 0.859	305.06 (±12.63); p = 0.385	324.9 (±12.37); p = 0.870	281.52 (±13.52)	276.25 (±13.15)	287.10 (±13.96)	283.92 (±13.71)	291.9 (±20.92)	284.50 (±22.03)	284.50 (±22.03)	291.9 (±20.92)
	ESR (mm/hr)	29.20 (±3.23); p = 0.318	27.13 (±2.91); p = 0.355	18.04 (±2.06); *p = 0.023	29.56 (±2.57); p = 0.953	25.12 (±2.88)	24.40 (±2.74)	29.60 (±3.07)	25.87 (±2.98)	15.20 (±2.85)	14.60 (±3.49)	14.60 (±3.49)	15.20 (±2.85)
	HsCRP (mg/L)	18.77 (±8.34); *p = 0.034	8.41 (±1.84); p = 0.066	4.59 (±0.81); *p = 0.007	9.84 (±1.46); p = 0.262	6.06 (±1.06)	6.72 (±1.08)	12.32 (±3.31)	10.34 (±3.34)	1.91 (±0.47)	1.59 (±0.51)	1.59 (±0.51)	1.91 (±0.47)
Weight	Weight (Kg)	68.60 (±1.25)	68.29 (±1.25)	67.02 (±1.22)	68.58 (±1.22)	67.02 (±1.78)	66.62 (±1.70)	68.50 (±1.61)	67.57 (±1.80)	64.20 (±4.07)	64.00 (±3.67)	64 (±3.67)	64.2 (±4.07)
	BMI	26.50 (±0.46)	26.39 (±0.48)	25.90 (±0.46)	26.52 (±0.47)	25.86 (±0.50)	25.70 (±0.45)	26.33 (±0.45)	26.07 (±0.49)	25.45 (±1.30)	25.39 (±1.16)	25.39 (±1.16)	25.45 (±1.3)

In Table 2, the serum creatinine levels in the STV group showed a slight improvement (decrease) by six months, and blood urea was significantly reduced (the STV group’s mean blood urea decreased at six months compared to baseline; p < 0.05). Although eGFR did not increase significantly, the stabilization (or slight improvement) of eGFR in the STV group contrasted with a tendency for eGFR to decline in the PLC group over six months (differences were not statistically significant but were directionally favorable for stevia).

Serum uric acid, which is often elevated as CKD progresses, significantly decreased in the STV group by the second follow-up (p < 0.01 vs. baseline), whereas it did not significantly change in the PLC group. This result is notable, as elevated uric acid is linked to renal function decline. The stevia-treated patients also showed improvements in microalbuminuria. At six months, the STV group’s urine microalbumin levels were significantly lower than baseline values (p < 0.003), while the PLC group showed no such improvement. Additionally, the ACR and PCR in urine showed a decreasing trend in the STV group, indicating reduced proteinuria, whereas these ratios remained unchanged or worsened in the PLC group over the same period. By the end of the trial, the STV group had significantly lower ACR and PCR compared to baseline, suggesting a renoprotective effect (these results are detailed in Table 3).

**Table 3:** Multiple comparisons among the biochemical variables of different treatment phases The data were analyzed by one-way ANOVA followed by Bonferroni’s test, and multiple comparisons were made. The stevia treatment group is expressed as STV (n = 43), the placebo treatment group as PLC (n = 40), and the healthy control group as CON (n = 10). Here, (**p < 0.01) = highly significant, (***p < 0.001) = very highly significant, and is considered as STV vs PLC. BL, Baseline Phase; 1st FF, 1st Follow-Up Phase; 2nd FF, 2nd Follow-Up Phase; WO, Washout Phase; UTP, Urinary Total Protein; eGFR, Estimated Glomerular Filtration Rate; ACR, Albumin-to-Creatinine Ratio; PCR, Protein-to-Creatinine Ratio

Dependent Variable	Treatment Phases	Treatment Groups	Treatment Groups	Std. Error	p-value	95% Confidence Interval	
						Lower Bound	Upper Bound
Blood Urea (mmol/L)	BL	STV	PLC	1.377	0.125	-0.51	6.21
			CON	2.201	0.066	-0.24	10.50
		PLC	STV	1.377	0.125	-6.21	0.51
			CON	2.217	0.916	-3.12	7.69
		CON	STV	2.201	0.066	-10.50	0.24
			PLC	2.217	0.916	-7.69	3.12
	1st FF	STV	PLC	0.717	1.000	-1.19	2.31
			CON	1.146	0.110	-0.37	5.23
		PLC	STV	0.717	1.000	-2.31	1.19
			CON	1.154	0.328	-0.95	4.68
		CON	STV	1.146	0.110	-5.23	0.37
			PLC	1.154	0.328	-4.68	0.95
	2nd FF	STV	PLC	0.2656	0.446	-0.261	1.035
			CON	0.4244	0.000***	0.844	2.915
		PLC	STV	0.2656	0.446	-1.035	0.261
			CON	0.4274	0.002**	0.450	2.535
		CON	STV	0.4244	0.000***	-2.915	-0.844
			PLC	0.4274	0.002**	-2.535	-0.450
	WO	STV	PLC	0.7800	0.160	-0.376	3.429
			CON	1.2466	0.011	0.673	6.755
		PLC	STV	0.7800	0.160	-3.429	0.376
			CON	1.2554	0.255	-0.875	5.250
		CON	STV	1.2466	0.011	-6.755	-0.673
			PLC	1.2554	0.255	-5.250	0.875
Serum Creatinine (mmol/L)	BL	STV	PLC	5.476	1.000	-14.53	12.19
			CON	8.751	0.001***	11.46	54.16
		PLC	STV	5.476	1.000	-12.19	14.53
			CON	8.813	0.001***	12.48	55.48
		CON	STV	8.751	0.001***	-54.16	-11.46
			PLC	8.813	0.001***	-55.48	-12.48
	1st FF	STV	PLC	5.766	0.288	-23.76	4.37
			CON	9.216	0.005**	7.16	52.12
		PLC	STV	5.766	0.288	-4.37	23.76
			CON	9.281	0.000***	16.70	61.98
		CON	STV	9.216	0.005**	-52.12	-7.16
			PLC	9.281	0.000***	-61.98	-16.70
	2nd FF	STV	PLC	5.449	0.203	-23.38	3.21
			CON	8.709	0.008**	5.82	48.31
		PLC	STV	5.449	0.203	-3.21	23.38
			CON	8.771	0.000***	15.75	58.54
		CON	STV	8.709	0.008**	-48.31	-5.82
			PLC	8.771	0.000***	-58.54	-15.75
	WO	STV	PLC	5.3739	0.476	-20.748	5.471
			CON	8.5885	0.002**	9.260	51.164
		PLC	STV	5.3739	0.476	-5.471	20.748
			CON	8.6490	0.000***	16.750	58.950
		CON	STV	8.5885	0.002**	-51.164	-9.260
			PLC	8.6490	0.000***	-58.950	-16.750
Microalbumin (SPOT) (mg/dL)	BL	STV	PLC	55.0057	0.696	-200.382	67.997
			CON	87.9097	0.482	-90.128	338.793
		PLC	STV	55.0057	0.696	-67.997	200.382
			CON	88.5296	0.102	-25.447	406.497
		CON	STV	87.9097	0.482	-338.793	90.128
			PLC	88.5296	0.102	-406.497	25.447
	1st FF	STV	PLC	58.810	1.000	-143.69	143.25
			CON	93.989	0.241	-63.12	395.46
		PLC	STV	58.810	1.000	-143.25	143.69
			CON	94.652	0.246	-64.52	397.30
		CON	STV	93.989	0.241	-395.46	63.12
			PLC	94.652	0.246	-397.30	64.52
	2nd FF	STV	PLC	46.751	1.000	-132.25	95.86
			CON	74.717	0.377	-66.75	297.80
		PLC	STV	46.751	1.000	-95.86	132.25
			CON	75.244	0.237	-49.84	317.28
		CON	STV	74.717	0.377	-297.80	66.75
			PLC	75.244	0.237	-317.28	49.84
	WO	STV	PLC	54.954	1.000	-178.28	89.85
			CON	87.828	0.294	-67.35	361.17
		PLC	STV	54.954	1.000	-89.85	178.28
			CON	88.447	0.100	-24.65	406.90
		CON	STV	87.828	0.294	-361.17	67.35
			PLC	88.447	0.100	-406.90	24.65
UTP (SPOT) (mg/dL)	BL	STV	PLC	16.582	1.000	-50.47	30.43
			CON	26.502	0.383	-23.90	105.41
		PLC	STV	16.582	1.000	-30.43	50.47
			CON	26.689	0.181	-14.33	115.88
		CON	STV	26.502	0.383	-105.41	23.90
			PLC	26.689	0.181	-115.88	14.33
	1st FF	STV	PLC	22.312	1.000	-40.07	68.79
			CON	35.658	0.377	-31.85	142.13
		PLC	STV	22.312	1.000	-68.79	40.07
			CON	35.910	0.778	-46.83	128.38
		CON	STV	35.658	0.377	-142.13	31.85
			PLC	35.910	0.778	-128.38	46.83
	2nd FF	STV	PLC	18.567	0.454	-18.42	72.17
			CON	29.674	0.177	-15.67	129.11
		PLC	STV	18.567	0.454	-72.17	18.42
			CON	29.883	0.962	-43.05	102.75
		CON	STV	29.674	0.177	-129.11	15.67
			PLC	29.883	0.962	-102.75	43.05
	WO	STV	PLC	19.367	1.000	-44.72	49.78
			CON	30.952	0.218	-19.29	131.73
		PLC	STV	19.367	1.000	-49.78	44.72
			CON	31.170	0.265	-22.35	129.73
		CON	STV	30.952	0.218	-131.73	19.29
			PLC	31.170	0.265	-129.73	22.35
Serum Uric Acid (mmol/L)	BL	STV	PLC	26.708	0.218	-16.62	113.69
			CON	42.684	1.000	-109.87	98.39
		PLC	STV	26.708	0.218	-113.69	16.62
			CON	42.985	0.630	-159.14	50.59
		CON	STV	42.684	1.000	-98.39	109.87
			PLC	42.985	0.630	-50.59	159.14
	1st FF	STV	PLC	23.626	1.000	-67.79	47.48
			CON	37.758	1.000	-115.02	69.21
		PLC	STV	23.626	1.000	-47.48	67.79
			CON	38.025	1.000	-105.51	80.01
		CON	STV	37.758	1.000	-69.21	115.02
			PLC	38.025	1.000	-80.01	105.51
	2nd FF	STV	PLC	19.907	0.001***	-123.64	-26.51
			CON	31.815	0.220	-135.29	19.94
		PLC	STV	19.907	0.001***	26.51	123.64
			CON	32.039	1.000	-60.76	95.56
		CON	STV	31.815	0.220	-19.94	135.29
			PLC	32.039	1.000	-95.56	60.76
	WO	STV	PLC	19.989	1.000	-35.27	62.26
			CON	31.947	1.000	-90.82	65.05
		PLC	STV	19.989	1.000	-62.26	35.27
			CON	32.172	1.000	-104.86	52.11
		CON	STV	31.947	1.000	-65.05	90.82
			PLC	32.172	1.000	-52.11	104.86
Urine for ACR (mg/mmol)	BL	STV	PLC	81.6247	1.000	-250.643	147.612
			CON	130.4519	0.523	-139.612	496.876
		PLC	STV	81.6247	1.000	-147.612	250.643
			CON	131.3717	0.250	-90.340	550.636
		CON	STV	130.4519	0.523	-496.876	139.612
			PLC	131.3717	0.250	-550.636	90.340
	1st FF	STV	PLC	65.004	1.000	-145.45	171.72
			CON	103.890	0.284	-78.02	428.87
		PLC	STV	65.004	1.000	-171.72	145.45
			CON	104.622	0.373	-92.94	417.52
		CON	STV	103.890	0.284	-428.87	78.02
			PLC	104.622	0.373	-417.52	92.94
	2nd FF	STV	PLC	64.0511	1.000	-159.017	153.495
			CON	102.3660	0.341	-86.176	413.278
		PLC	STV	64.0511	1.000	-153.495	159.017
			CON	103.0877	0.331	-85.175	417.800
		CON	STV	102.3660	0.341	-413.278	86.176
			PLC	103.0877	0.331	-417.800	85.175
	WO	STV	PLC	85.278	0.309	-348.53	67.55
			CON	136.290	0.642	-161.91	503.07
		PLC	STV	85.278	0.309	-67.55	348.53
			CON	137.251	0.077	-23.76	645.90
		CON	STV	136.290	0.642	-503.07	161.91
			PLC	137.251	0.077	-645.90	23.76
Urine for PCR (mg/mmol)	BL	STV	PLC	0.1903	1.000	-0.522	0.406
			CON	0.3041	0.244	-0.206	1.278
		PLC	STV	0.1903	1.000	-0.406	0.522
			CON	0.3062	0.167	-0.154	1.341
		CON	STV	0.3041	0.244	-1.278	0.206
			PLC	0.3062	0.167	-1.341	0.154
	1st FF	STV	PLC	9.5523	0.832	-12.863	33.744
			CON	15.2665	1.000	-26.433	48.053
		PLC	STV	9.5523	0.832	-33.744	12.863
			CON	15.3741	1.000	-37.136	37.875
		CON	STV	15.2665	1.000	-48.053	26.433
			PLC	15.3741	1.000	-37.875	37.136
	2nd FF	STV	PLC	9.55451	0.936	-13.5963	33.0212
			CON	15.26995	1.000	-27.1548	47.3487
		PLC	STV	9.55451	0.936	-33.0212	13.5963
			CON	15.37761	1.000	-37.1299	37.8989
		CON	STV	15.26995	1.000	-47.3487	27.1548
			PLC	15.37761	1.000	-37.8989	37.1299
	WO	STV	PLC	0.39991	0.000***	-2.8402	-0.8891
			CON	0.63913	1.000	-1.0198	2.0985
		PLC	STV	0.39991	0.000***	0.8891	2.8402
			CON	0.64364	0.001***	0.8338	3.9742
		CON	STV	0.63913	1.000	-2.0985	1.0198
			PLC	0.64364	0.001***	-3.9742	-0.8338
eGFR (mL/min/1.73m^2^)	BL	STV	PLC	4.251	1.000	-9.02	11.72
			CON	6.794	0.000***	-57.35	-24.20
		PLC	STV	4.251	1.000	-11.72	9.02
			PLC	6.842	0.000***	-58.82	-25.43
		CON	STV	6.794	0.000***	24.20	57.35
			PLC	6.842	0.000***	25.43	58.82
	1st FF	STV	PLC	4.558	1.000	-7.42	14.82
			CON	7.285	0.000***	-55.09	-19.55
		PLC	STV	4.558	1.000	-14.82	7.42
			CON	7.336	0.000***	-58.92	-23.13
		CON	STV	7.285	0.000***	19.55	55.09
			PLC	7.336	0.000***	23.13	58.92
	2ndFF	STV	PLC	4.366	0.678	-5.33	15.97
			CON	6.978	0.000***	-52.60	-18.55
		PLC	STV	4.366	0.678	-15.97	5.33
			CON	7.027	0.000***	-58.04	-23.76
		CON	STV	6.978	0.000***	18.55	52.60
			PLC	7.027	0.000***	23.76	58.04
	WO	STV	PLC	4.215	1.000	-7.29	13.28
			CON	6.737	0.000	-55.84	-22.97
		PLC	STV	4.215	1.000	-13.28	7.29
			CON	6.784	0.000***	-58.95	-25.85
		CON	STV	6.737	0.000***	22.97	55.84
			PLC	6.784	0.000***	25.85	58.95

Notably, stevia supplementation was associated with reductions in systemic inflammation markers. hsCRP levels dropped significantly in the STV group by six months (from elevated baseline levels to a significantly lower mean; p < 0.01 for change). Similarly, ESR, another inflammation indicator, decreased significantly in the STV group by the second follow-up (p < 0.05). In contrast, the PLC group did not show significant changes in hsCRP or ESR.

These findings suggest an anti-inflammatory effect of stevia in CKD patients. However, during the washout period (when stevia was stopped from months 7 to 9), both hsCRP and ESR levels in the STV group tended to rise again, indicating that continuous stevia intake might be required to sustain its anti-inflammatory benefits.

**Figure 2:**
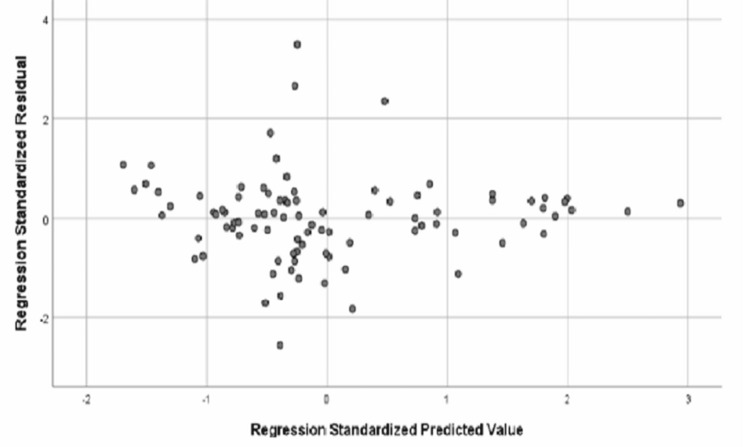
Scatter plot from the regression analysis showing the relationship between baseline eGFR and urinary parameters across different treatment phases Scatter plot showing the dependent variable: baseline eGFR (mL/min/1.73 m²). eGFR, Estimated Glomerular Filtration Rate

**Figure 3:**
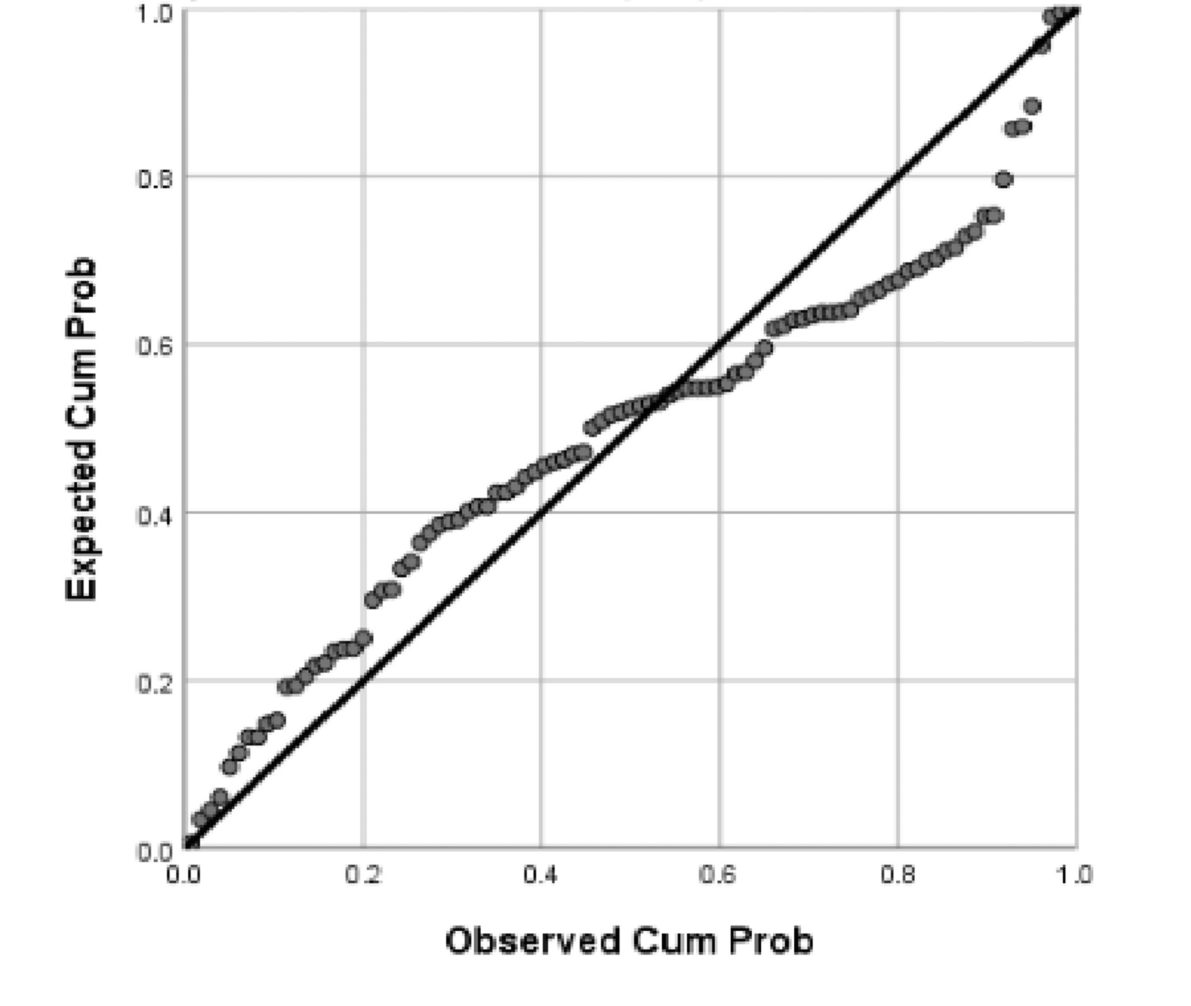
Normal P-P (probability-probability) plot of the standardized residuals from the regression analysis for baseline eGFR, comparing observed versus expected cumulative probabilities Normal P-P plot of regression standardized residuals for the dependent variable: baseline eGFR, measured in mL/min/1.73 m². eGFR, Estimated Glomerular Filtration Rate

To further explore the relationships among variables, regression analyses were performed. We examined whether baseline kidney function (eGFR at baseline) was associated with changes in urinary biomarkers over time. Scatter plots and linearity plots from these regression analyses are presented in Figures 2-3. The regression analysis considered baseline eGFR as the dependent variable and various urinary variables (e.g., ACR, PCR, and microalbumin) at different treatment phases as independent predictors. The scatter and linear plots of the regression analysis showed an association between the dependent variable (baseline eGFR) and the urinary variables across the different treatment phases (Figures 2-3). In general, these plots help visualize how baseline renal function might correlate with urinary output measures, and whether the relationships hold across treatment periods.

In this scatter plot (Figure 2), the regression residuals versus predicted values illustrate the relationship between baseline eGFR (the dependent variable) and various urinary parameters at different treatment phases. Each point represents an individual patient’s data. The pattern of points suggests how well the regression model correlates baseline kidney function with the urinary biomarkers. A tighter clustering of points around a diagonal line would indicate a strong association. In this plot, there is some spread, implying a moderate correlation: patients with higher baseline eGFR tend to have correspondingly favorable levels of urinary markers across phases, though variability exists. The scatter does not show gross heteroscedasticity or outliers, supporting the validity of the regression model used for analysis.

In Figure 3, a normal P-P plot of the regression standardized residuals for baseline eGFR is presented. The observed cumulative probability of the residuals is plotted against the expected cumulative probability if the residuals followed a perfect normal distribution. The points in the plot lie reasonably close to the diagonal reference line, indicating that the residuals of the regression are approximately normally distributed. This suggests that the linear regression model’s assumptions are met and that the model provides a good fit for the data. In practical terms, the P-P plot supports the reliability of the regression findings by confirming that there are no major deviations from normality in the residuals, which could otherwise indicate model misspecification.

Multiple comparison tests (post-hoc analyses) were conducted to compare changes between groups at each time point (detailed in Table 3). Key findings include significant improvements in the STV group at the second follow-up (p < 0.000). A decrease in serum creatinine levels was significant at the first follow-up between the STV and CON groups (p < 0.005), and again at the washout phase (p < 0.002). Serum uric acid levels also showed significant differences at the second follow-up between the STV and PLC groups (p < 0.001). Similarly, eGFR showed significant differences at multiple time points: at baseline between STV and CON (p < 0.000), at the first follow-up (p < 0.000), and at the second follow-up (p < 0.000). Overall, stevia supplementation for six months led to significant improvements in blood pressure, blood glucose, renal filtration markers (blood urea and serum uric acid), and inflammation markers (hsCRP) in CKD patients - beyond any changes observed with placebo. The washout results indicate that many of these beneficial effects were at least partly reversible when stevia was discontinued, underscoring the importance of ongoing treatment for sustained benefits.

## Discussion

The increased prevalence of CKD has attracted global attention due to its economic burden on patients, family members, and society. Research has found that oral intake of stevioside by hypertensive, non-diabetic individuals demonstrates a prolonged blood pressure-lowering and antihyperglycemic effect [18]. Therefore, the current study investigated whether chronic administration of stevioside could prevent the progression of CKD sufficiently to justify its use as a new treatment option. To our knowledge, this is the first clinical study on CKD patients in Bangladesh, and some of its preliminary results (baseline and first follow-up) were published earlier, showing that stevia demonstrated an improving trend in renal parameters among CKD patients at Stage I-III [16]. To preserve renal function in individuals with CKD, early diagnosis and appropriate therapy should be prioritized [18].

Renin-angiotensin-aldosterone system (RAAS) blockers, such as ACE inhibitors and/or ARBs, have been proven beneficial for proteinuric CKD patients and are widely prescribed by physicians [18]. The eighth report of the Joint National Committee on the management of high blood pressure in adults recommended that CKD patients, whether younger or older than 60 years of age, should maintain a blood pressure below 140/90 mmHg, and encouraged the use of ACE inhibitors or ARBs to treat hypertension in CKD patients - regardless of ethnic background - either as first-line therapy or in addition to it [19]. The report also showed that patients with concomitantly very high or very low SBP and DBP had the highest fatality rates [18].

Our study showed that stevia significantly reduced blood glucose levels and SBP in CKD patients after the first and second follow-ups; interestingly, SBP increased back to baseline levels during the washout period when no stevia was given. These outcomes support other studies that demonstrated stevioside’s anti-hyperglycemic, anti-hypertensive, anti-inflammatory, and anti-cancer effects [20]. A possible mechanism of stevioside action was reported to increase insulin secretion and improve glucose uptake in peripheral tissues by enhancing insulin sensitivity [21]. Additionally, reports on experimental animals demonstrated that stevia’s anti-hypertensive effect was mediated by blocking L-type calcium channels [22,23].

In the current study, stevia showed a beneficial effect in CKD patients by improving microalbuminuria. Treatment with stevioside also significantly reduced blood urea and slightly improved serum creatinine levels at the end of the second follow-up treatment period. A probable explanation might be stevia’s antioxidant properties, which help reduce metabolic and cardiovascular problems since oxidative stress and inflammation are linked to diabetes, cardiovascular disease, and the progression of CKD [12]. Moreover, stevioside showed an inhibitory effect on glucagon secretion, altering gluconeogenesis and glycogenolysis. The nephroprotective effect of stevia might be due to enhanced superoxide dismutase (SOD) and catalase levels or decreased glucose levels, which are associated with reductions in serum creatinine and urea levels. Additionally, stevia was found to reduce the formation of reactive oxygen species (ROS) and glycosylated end products and demonstrated free radical scavenging activity [21]. Hence, these multimodal mechanisms of stevia, including calcium channel blocking activity, might contribute to its renoprotective effects in CKD patients [22].

Cross-sectional studies on CKD prevalence conducted in India revealed that rural communities demonstrated the lowest prevalence of proteinuria (2.25%) compared to the urban population, which had a higher frequency (4.41%), contributing to a high death rate [24]. Because of the high mortality rate, Singh et al. recommended that patients with proteinuria should be appropriately managed [24]. We previously showed stevia’s beneficial effects on the management of serum albumin levels and renal parameters in an animal model of nephrotoxicity [16]. The current study showed that the PCR and ACR ratios significantly decreased in stevia-treated CKD patients. 

Declining glomerular filtration rate (GFR) is a significant endpoint for CKD patients, leading to increased serum creatinine and uric acid levels [25]. An eGFR of less than 60 mL/min/1.73 m² and a urinary ACR of 1.13 mg/mmol or more are independent predictors of mortality risk in the general population [25]. Studying the role of serum uric acid in CKD is difficult, since uric acid is excreted primarily by the kidneys, and a decrease in GFR is accompanied by a rise in serum uric acid levels. However, a recent Japanese study assessed the impact of serum uric acid on the natural history of eGFR. The authors claimed that if the elevation of serum uric acid is a result, rather than a cause, of declining eGFR, the relationship between serum uric acid and eGFR should remain consistent within the same population over the years - except for shifts due to age-dependent reductions in eGFR [26]. According to some research, serum uric acid itself may be detrimental to CKD patients by causing inflammation that promotes CKD progression [27]. Although still debated, these findings are supported by large prospective observational studies indicating that increasing serum uric acid levels predict the development and progression of CKD in many populations [27].

The current study showed a significant decrease in serum uric acid after the first and second follow-ups when stevia was given to CKD patients; however, an insignificant increase in eGFR was observed among the participants after six months of stevia intake. In this study, the reduction of serum uric acid after stevia intake could be considered an important predictor supporting stevia’s ability to improve renal function and slow CKD progression. Serum uric acid increase was associated with worsening eGFR and ACR over time, and these two markers independently predict advanced-stage CKD, cardiovascular disease, and mortality [28,29]. Numerous reports suggest serum uric acid is a risk factor for CKD and cardiovascular disease [30,31]. After adjustment for baseline eGFR, a slightly elevated uric acid level (7-8.9 mg/dL) was associated with a nearly doubled risk of incident CKD. This increased risk remained significant even after adjustment for baseline eGFR, gender, age, antihypertensive drugs, and components of the metabolic syndrome [31]. Weiner et al. mentioned that an increase of 1 mg/dL in serum uric acid was associated with a 7%-11% increase in the incidence of CKD, where proteinuria and allopurinol were used [32]. Moreover, high uric acid levels favor proinflammatory mechanisms by enhancing the production of inflammatory markers.

CKD patients were found to be associated with inflammation, which contributes to the progressive deterioration of renal parameters. Alarmingly, chronic inflammation plays a role in both the causes and consequences of different forms of CKD [15]. Current knowledge links CKD progression with injured tubular epithelial cell (TEC)-mediated inflammation and altered glycolysis, involving 6-phosphofructo-2-kinase/fructose-2,6-bisphosphatase-3 (PFKFB-3), resulting in increased lactate production [33]. PFKFB-3-mediated glycolysis remains intertwined with inflammation via activation of nuclear factor kB (NF-κB) and contributes to kidney fibrosis [34]. Considering the beneficial effects of stevia in metabolic disorders and diabetes, the stevia-mediated reduction of hsCRP and ESR observed in the current study among CKD patients may suggest a potential interaction with PFKFB-3. Further research is needed to determine whether stevia’s ability to target PFKFB-3 could open a new avenue in the treatment of CKD.

Limitations

One key limitation of our study is the short duration for assessing critical outcomes related to CKD progression, such as doubling of creatinine levels or decline to ESRD. While nine months allowed us to observe changes in surrogate markers like blood pressure and ACR, longer trials are essential to confirm effects on clinical endpoints, including time to Stage IV CKD or dialysis. Additionally, although our sample size (n = 83 CKD patients) was adequate to detect moderate effects in lab parameters, a larger trial would better capture variability and subgroup responses. The CON group (n = 10) was also small, primarily serving to establish reference ranges. Future studies should include a larger control group or an active comparator, such as another supplement, to more effectively validate the effects of stevia.

## Conclusions

Stevia’s multifaceted benefits might offer a novel means to alleviate cardiovascular and metabolic risks in early CKD. While stevia is not a replacement for standard CKD treatments, it could serve as a safe, supplementary approach. Further long-term studies and clinical trials with larger cohorts are warranted to confirm these findings and to determine whether stevia can translate into slower progression to advanced CKD or improved patient outcomes, such as reduced incidence of dialysis or CKD-related cardiovascular events. Additionally, investigating optimal dosing, long-term safety, and its effects in later-stage CKD would be valuable. In conclusion, incorporating stevia as a therapeutic supplement may open a new avenue in holistic CKD management - especially beneficial for patients in early stages aiming to preserve renal function and reduce risk factors. Further research is needed to confirm the claim and to find out the molecular mechanisms of stevia in CKD.

## Appendices

Questionnaire of the study

Name of the Patient/Participant: ____________

Date: ___________

Hospital ID: ___________

Address of the Patient/Participant: ____________

Mobile/Phone No.: _____________

(A) Socio-Demographic Profile

1. Age: ________ Yrs

2. Gender: Male/Female

3. Height: ____ cm ____ft ____inch

4. Weight: ____ kg

5. BMI: ____ m^2^/kg

6. Marital Status

  Single

  Married

  Divorced

  Widow

7. Occupation

  Business

  Service

  Housewife

  Other

8. Educational Status

  Primary Education

  Secondary School Certificate (SSC)

  Higher Secondary School Certificate (HSC)

  Graduate

  Postgraduate

  Above

9. Monthly Income

  <10,000.00

  10,000 to <20,000.00

  20,000 to <30,000.00

  30,000 to <40,000.00

  40,000 to <50,000.00

  >50,000.00

10. No. of Family members

  1=2

  2=3

  3=4

  4=5

  5=6

  6=7

  8=More

11. Affected family member

  1=1

  2=2

  3=3

  4=More

(B) Clinical Investigation

1. Time of 1st visit: _________

2. Weight on-

  Baseline ____ kg

  3rd Month ____ kg

  6th Month ____ kg

  Wash Out ____ kg

3. Cause of CKD (Chronic Kidney Disease): ________

4. Year of Diagnosis ________

5. Systolic Blood Pressure (SBP) and Diastolic Blood Pressure (DBP) on,

  Baseline: SBP___ / DBP ___

  3rd Month: SBP___ / DBP ___

  6th Month: SBP___ / DBP ___

  Wash Out: SBP___ / DBP ___

6. Cause of renal diseases:

  Hypertension,

  Diabetes mellitus,

  Chronic Nephritis,

  Others

7. History of Smoking:

  Yes

  No

8. Alcohol intake

  Yes

  No

9. Nutritional Status:

  (I) MUAC on,

     Baseline

     3rd Month

     6th Month

     Wash Out

  (II) nPNA (Normalized Protein Nitrogen Appearance) on,

     Baseline

     3rd Month

     6th Month

     Wash Out

(C) Laboratory Investigation

**Table 4:** Laboratory investigation

Sl No.	Investigations	Results			
		Baseline	3rd Month	6th Month	Wash Out
		Date:	Date:	Date:	Date:
1	Blood Urea				
2	Serum Creatinine				
3	Electrolytes:				
	(i) Na				
	(ii) K				
	(iii) Cl				
	(iv) TCO2				
	(v) Inorganic Phosphate				
4	Serum Total Protein				
5	Microalbumin (Spot)				
6	Urinary Total Protein (UTP) (SPOT)				
						7	Serum Uric acid				
8	(i) Blood Sugar - Fasting (FBS)				
						(ii) Blood Sugar - Postprandial (PBS)				
	9	Urine for ACR (Albumin: Creatinine)									
	10	Urine for PCR (Protein: Creatinine)				
11	eGFR.				
12	Hemoglobin				
13	Total Count (TC) of WBC				
14	Differential Count of WBC:				
	(i) Monocyte				
	(ii) Neutrophil				
	(iii) Eosinophil				
	(iv) Basophil				
15	Total Count of RBC				
16	RBC indices:				
	(i) Hematocrit (HCT)				
	(ii) Mean Corpuscular Volume (MCV)				
	(iii) Mean Corpuscular Hemoglobin (MCH)				
	(iv) Red-Cell Distribution Width-CV (RDW-CV)				
17	Total Count of Platelets				
	Erythrocyte Sedimentation Rate (ESR)				
18	Serum Total Protein (STP)				
19	High Sensitive C-Reactive Protein (HsCRP)				
20	Cholesterol				
21	Triglyceride				
22	High Density Lipoprotein (HDL)				
						23	Low Density Lipoprotein (LDL)				

Signature by the PhD Candidate: _____________ 

Date: ___________

Signature by the Clinical Investigator: ___________

Date: ___________
